# Comparison of intestinal parasitic infection in newly arrived and resident workers in Qatar

**DOI:** 10.1186/1756-3305-4-211

**Published:** 2011-11-04

**Authors:** Marawan A Abu-Madi, Jerzy M Behnke, Ahmed Ismail, Nada Al-Olaqi, Kefah Al-Zaher, Roda El-Ibrahim

**Affiliations:** 1 Department of Health Sciences, College of Arts & Sciences, Qatar University, P.O. Box 2713, Doha, Qatar; 2 School of Biology, University of Nottingham, University Park, Nottingham, NG7 2RD, UK; 3 Medical Commission Department, Supreme council of Health, Qatar; 4 Department of Laboratory Medicine and Pathology, Hamad Medical Corporation, Qatar

**Keywords:** Qatar, intestinal helminths, intestinal protozoa, residents, recent immigrants

## Abstract

**Background:**

The rapid growth of Qatar in the last two decades has been associated with an enormous expansion of building programs in its cities and in the provision of new service industries. This in turn has attracted a large influx of immigrant workers seeking employment in jobs associated with food handling, domestic service and the building industry. Many of these immigrants come from countries in the tropics and subtropics where intestinal parasitic infections are common.

**Methods:**

We analyzed intestinal parasitic infections recorded in 2008 among immigrant and long-term resident workers in Doha city, Qatar (*n *= 1538). Stool examinations were carried out at the Hamad Medical Corporation and at the Medical Commission in Doha using standard procedures.

**Results:**

Overall, 21.5% of subjects were infected with at least one of the species recorded (8 helminth and 4 protozoan species; the highest prevalence was for hookworms = 8.3%) and there were strong regional effects on prevalence of helminths, with subjects from North East Africa and Nepal showing particularly high prevalence. Most helminths declined in prevalence in subjects that acquired residency status in Qatar, especially among female subjects, but there was a marked exception among male Nepalese workers, who continued to harbour helminth infections (notably hookworms) after they became residents. Contrary to all other regional groups the prevalence of *Giardia duodenalis *was higher among Nepalese residents compared with new arrivals, while *Blastocystis hominis *infections were more common among residents of all regions, and especially among North East Africans.

**Conclusions:**

Our analysis has identified male Nepalese workers as a particular risk group continuing to harbour hookworm infection and *G. duodenalis *as residents, and subjects from North East Africa are as particularly likely to acquire *B. hominis *infection after settling in the country. These conclusions have important implications for the health authorities in Qatar.

## Background

The rapid growth of Qatar in the last two decades has been associated with an enormous expansion of building programs in its cities, notably in Doha, and in the provision of new service industries. This in turn has attracted a large influx of immigrant workers seeking employment in jobs associated with food handling, domestic service and the building industry. Many of these immigrants come from countries in the tropics and subtropics where intestinal parasitic infections are common [1-3].

Immigrants seeking work in Qatar are examined by the Medical Commission on arrival and are now routinely given albendazole as a condition of entry, residence and issuance of a work permit. This treatment is administered within 7-10 days of arrival, but there is no follow-up, other than for those seeking employment in the food handling industry, who have to undergo annual check-ups. In earlier studies [4] seven species of intestinal parasites were identified as relatively common among newly arrived immigrants. These included three nematode species (*Trichuris trichiura*, hookworms and *Ascaris lumbricoides*) and four protozoans (*Entamoeba histolytica*/*dispar*, non-pathogenic *Entamoebae*, *Blastocystis hominis *and *Giardia duodenalis*). The overall prevalence of infections with all species combined was as high as 33.9% (13.6% for nematodes and 24.8% for protozoa) but this varied significantly depending on the country of origin and some individuals (0.1%) harboured as many as 5 species concurrently. A major determinant of the type and intensity of parasitic infection carried into Qatar by new immigrants was their country of origin [4].

The treatment administered within a short period of arrival should prevent the dissemination of helminths among the local population in Qatar, but little is known about the persistence of infections among long-term residents, and immigrants who have settled in the country. A recent study, covering 28 nationalities now resident in Qatar, found that 10.2% of those examined were infected with at least one species of intestinal parasite (2.6% with helminths and 8.0% with protozoan species). The majority of helminth infections (69%) were caused by hookworms, which were mainly found in 20-30 year-old male subjects from Nepal. The remaining cases were mostly among Asian immigrants from bordering countries but a worrying trend with respect to both helminth and protozoan parasites was the increase in prevalence over the period 2005-2008.

Although these two studies focused on immigrants to Qatar, the former examining recent arrivals and the latter examining long-term residents, to-date there has been no direct comparison of these two groups. Therefore, in the present work, we examined two independent data-sets, held at The Hamad Medical Corporation (HMC) for long-term residents and The Medical Commission for recent arrivals, in order to obtain an objective assessment of the extent to which prevalence of parasitic infections differs between these two groups. The results of this analysis will inform the local health authorities, facilitating better implementation of remedial action for limiting transmission and dissemination of parasitic infections in the country. Because workers from Asia in general and young adult male Nepalese workers in particular, have been identified as the major source of helminth infections among long-term residents in Qatar [5], Nepalese immigrants were analyzed separately from other groups.

## Methods

### Selection of subjects, sample collection and stool examination

The present study was based on a cross-sectional survey of intestinal parasitic infections among recently arrived immigrants in specific jobs (food handlers and housemaids) and long-term residents, who had been referred to the Medical Commission and Microbiology Laboratory at HMC respectively for the routine stool test during the period between June-September 2009. Random stool samples were collected from labourers at the Medical Commission soon after arrival in the country, during their participation in the routine health examination, as previously described [4]. Samples from the long-term resident subjects were not a completely random collection or an entirely unbiased selection by virtue of their referral for consultation at the hospital. Nevertheless, the data-base did provide an opportunity to compare parasitic infections across a large age range, from both sexes and from a range of nationalities. Data from a subject were used only if the subject was originally from a country that was represented among both the residents and recent immigrants, and was at least 18 years of age (working age). Therefore, after excluding 468 subjects from countries where all available records were only for residents (16 countries, among which 258 records were for Qataris, 42 for subjects from Pakistan, 29 from Jordan, 21 from Palestine, 22 from Iran, 21 from Yemen and for the remaining countries fewer than 20 records each), we used all the remaining available records that met the inclusion criteria. Stool examination was carried out in a safety cabinet, where each stool specimen was preserved in an Ecofix preservative vial (Meridin Biosciences, Inc.) in which in the majority of cases, the samples were fixed within 1-2 hours of collection. The contents were mixed vigorously by vortex and the homogenized stool sample was kept for half an hour at room temperature to ensure adequate fixation. The preserved specimen was mixed by vortex and filtered through a macro-con filtration unit for the removal of bulky debris. After filtration, 10% formalin and ethyl acetate were added; the sample was centrifuged for 10 min at 3000 rpm and the fluid containing diethyl ether and formalin was discarded. The pellet was re-suspended by agitation, poured onto a microscope slide containing one drop of iodine and examined microscopically for the identification of parasite eggs/cysts. Amoeba species other than *E. histolytica/dispar *including *E. coli*, *E. hartmanii*, *Endolimax nana *and *Iodamoeba butschlii *were pooled together and recorded as non-pathogenic amoebae because the cysts were indistinguishable [4] and we refer to *Giardia duodenalis *(= *lamblia/intestinalis*) [5]. Ethical approval for access to these data was obtained from the Medical Research Centre and the Research Committee at HMC, Qatar (Research protocol # 8060/09).

### Definition of variables

All birth dates and examination dates were recorded meticulously and the ages of subjects were classified into ranges by years. Seven age classes were then constructed to span 18.0-22.9 (age class 1, *n *= 184), 23.0-26.9 (age class 2, *n *= 326), 27.0-30.9 (age class 3, *n *= 254), 31.0-35.9 (age class 4, *n *= 274), 36.0-40.9 (age class 5, *n *= 212), 41.0-45.9 (age class 6, *n *= 139) and ≥ 46 years (age class 7, *n *= 149).

The subjects in this study came from 11 countries. For the purpose of analysis, the subjects were grouped into four geographical regions of origin, with Nepal as a separate category. Nepal was treated separately because earlier published data had indicated that parasitic infections were particularly prevalent among workers originating from this country [5]. The groupings were as follows (constituent countries and number of subjects are given in parenthesis): North Africa (Egypt, *n *= 182; Morocco, *n *= 7; Tunisia, n = 15), North East Africa (Ethiopia, *n *= 157; Sudan, *n *= 63), Western Asia (Bangladesh, *n *= 66; India, *n *= 248; Sri Lanka, *n *= 122), Eastern Asia (Indonesia, *n *= 151; Philippines, *n *= 291) and Nepal (*n *= 236).

### Statistical Analysis

Analysis of age was conducted by a 3-factor general linear model (GLM), with age as the dependent variable, and sex (2 levels, male and female), region of origin (5 levels, the 5 regions of origin) and residency status (2 levels, new arrivals and resident) as fixed factors, in SPSS (version 16.0.0). Prevalence data (percentage of subjects infected) are shown with 95% confidence intervals (in columns in tables, in parenthesis in the text and as error bars on figures) employing bespoke software [6]. Prevalence was analyzed by maximum likelihood techniques based on log linear analysis of contingency tables using the software package SPSS (Version 16.0.0.). Initially, full factorial models were fitted, incorporating as factors sex (2 levels, males and females), age (7 levels as given above), residency status (resident or recent immigrant) and region of origin (5 levels, as defined above). Infection was considered as a binary factor (present/absent). These explanatory factors were fitted initially to all models that were evaluated. For each level of analysis in turn, beginning with the most complex model, involving all possible main effects and interactions, those combinations that did not contribute significantly to explaining variation in the data were eliminated in a stepwise fashion beginning with the highest-level interaction (backward selection procedure). A minimum sufficient model was then obtained, for which the likelihood ratio (LR) of χ^2^ was not significant, indicating that the model was sufficient in explaining the data (some of these values are given in the legend to the figure). The importance of each term (i.e. interactions involving infection) in the final model was assessed by the probability that its exclusion would alter the model significantly and these values relating to interactions that included presence/absence of infection are given in the text. The remaining terms in the final model that did not include presence/absence of infection, and the LR of χ^2 ^for final models that are not given, can be made available from the authors on request.

Factors that may have caused aggregation of multiple infections within groups of people were examined by the loglinear analysis described above but confined to the subjects harbouring more than one species of parasite. Possible associations between species were compared to null models based on prevalence [7].

## Results

### Study group

Table 1 gives further details on the composition of the study group, indicating the numbers of each sex within the geographical groupings and the average age of residents versus recent immigrants within each region. The number of subjects in most cells in the table was adequate for analysis, but three cells were under-represented (only 1 female newly arrived immigrant from North Africa, and two females from Nepal in each of the two residency classes). These discrepancies are taken into account in the analysis.

**Table 1:** Distribution of subjects by region, residency class and sex, and the mean ages of subjects within factor levels.

Country*	No. new immigrants		No. residents		Total	Mean age**(n)				
			
	Males	Females	Males	Females		New immigrants		Residents		Combined
N. Africa	82	1	94	27	204	30.1	(83)	37.3	(121)	34.3 (204)
N.E. Africa	48	101	32	39	220	25.8	(149)	36.3	(71)	29.2 (220)
W. Asia	62	74	244	56	436	32.6	(136)	36.6	(300)	35.3 (436)
E. Asia	141	204	71	26	442	30.3	(345)	38.1	(97)	32.0 (442)
Nepal	51	2	181	2	236	24.7	(53)	29.3	(183)	28.2 (236)
**TOTAL**	**384**	**382**	**622**	**150**	**1538**					
**COMBINED**						**29.4**	**(766)**	**35.1**	**(772)**	**32.3 (1538)**

*N. Africa comprised Egypt & Morocco; N. E. Africa, Ethiopia & Sudan; W. Asia, Bangladesh, India & Sri Lanka; E. Asia, Indonesia & Philippines.

** age is given in years

The age range of the study group was 18-52 years with a mean of 32.3 ± 0.22. However, analysis indicated that there were significant differences between residents and recent immigrants and also between subjects from the five regions. The most significant discriminating factor was residency status (3-factor GLM, with residency status, sex and region of origin as factors on age, for main effect of residency status *F*_1,1523 _= 204.2, *P *< 0.001). However, as can be seen in Table 1, newly arrived immigrants were on average 5.7 years younger than residents, so although the finding was statistically significant in biological terms the effect is likely to be quite small. The difference in age between subjects from different geographic regions was also significant although statistically much weaker than the effect of residency status (main effect of region of origin, *F*_4,1523 _= 8.8, *P *< 0.001). Subjects from Nepal were on average 7.1 years younger than those from W. Asia, with other regions fitting between these, so again a relatively small difference, with probably little biological significance. There was no difference between the sexes, but there were two significant interactions (sex*region of origin, *F*_4,1523 _= 7.3, *P *< 0.001 and region of origin*residency status *F*_4,1523 _= 4.7, *P *= 0.001).

### All parasite species combined

Overall 21.5% of the subjects in this study carried at least one of the parasites (helminth and/or protozoan) (Table 2). The strongest significant effect on prevalence was that of region of origin (Table 3) with subjects from N.E. Africa and from Nepal showing higher prevalence of parasitic infections compared to those from N. Africa and W. Asia, while those from E. Asia were intermediate (χ^2^_4 _= 27.4, *P *< 0.001). Residency status also had a marked effect on prevalence, but was dependent on host sex (for sex*residency status*presence/absence of parasites χ^2^_1 _= 11.8, *P *= 0.001). Both sexes of new immigrants showed very similar prevalence (females = 28.0% [22.96-33.69] and males = 25.0% [20.21-30.51]), and prevalence was lower in both sexes among residents, but among the latter, prevalence of infection was very low among females (6.7% [3.51-12.19]) but 2.8 fold higher among resident males (18.8% [16.25-21.63]).

**Table 2:** Prevalence of all species and higher taxa in the whole study group and by residency status

Species	New immigrants		Residents		Combined	
	
	%	95% CI	%	95% CI	%	95%CI
**Protozoa**						
*B. hominis*	0.8	0.36 - 1.77	5.1	3.61 - 6.99	2.9	2.13 - 3.92
*G. duodenalis*	2.9	1.82 - 4.44	1.6	0.86 - 2.82	2.2	1.53 - 3.09
Amoeba non path	3	1.92 - 4.60	3.2	2.11 - 4.88	3.1	2.30 - 4.14
Amoeba path	1.3	0.68 - 2.50	0.3	0.12 - 0.97	0.8	0.40 - 1.36
All protozoa combined	7.4	5.65 - 9.70	8.8	6.86 - 11.23	8.1	6.76 - 9.49
**Helminths**						
Hookworms	10.3	8.17 - 12.87	6.2	4.57 - 8.33	8.3	6.88 - 9.63
*Trichuris trichiura*	6	4.39 - 8.08	1	0.49 - 2.16	3.5	2.64 - 4.58
*Ascaris lumbricoides*	3.3	2.14 - 4.90	0.6	0.30 - 1.57	2.5	1.80 - 3.47
*Strongyloides stercoralis*	0	0 - 0.57	0.9	0.42 - 1.97	0.5	0.18 - 0.94
*Enterobius vermicularis*	0	0 - 0.57	0.1	0.06 - 0.76	0.1	0 - 0.36
Hymenolepis nana*	1.6	0.87 - 2.83	0.4	0.18 - 1.17	1	0.55 - 1.61
*Taenia *spp.	0.7	0.30 - 1.57	0.4	0.18 - 1.17	0.5	0.22 - 1.02
*Schistosoma *spp.	0	0 - 0.57	0.3	0.12 - 0.97	0.1	0.02 - 0.47
All helminths combined	20	17.08 - 23.19	9.2	7.20 - 11.66	14.6	12.80 - 16.33
All the above species combined	26.5	23.26 - 30.00	16.5	13.79 - 19.49	21.5	19.40 - 23.51

*This species is also known as *Rodentolepis nana*

**Table 3:** Prevalence of the more common species by region of origin.

	N. Africa		N.E. Africa		W. Asia		E. Asia		Nepal	
Parasites	%	95%CI	%	95%CI	%	95%CI	%	95%CI	%	95%CI
All species	**14.7**	11.80-18.11	**29.1**	25.08-34.44	**16.5**	12.17-21.85	**21.3**	16.32-27.01	**29.7**	25.51-34.15
Helminths	**6.9**	4.87-9.44	**19.1**	15.75-22.95	**11.2**	7.66-15.98	**14.3**	10.20-19.35	**23.7**	19.91-27.98
Hookworms	**2**	1.04-3.63	**10.5**	7.90-13.61	**6**	3.54-9.77	**7.2**	4.51-11.37	**17.8**	14.40-21.73
*T. trichiura*	**2**	1.04-3.63	**3.2**	1.88-5.19	**3.2**	1.54-6.35	**5.7**	3.30-9.42	**1.7**	0.83-3.43
*A. lumbricoides*	**2**	1.04-3.63	**2.3**	1.23-4.09	**1.6**	0.56-4.25	**1.8**	0.66-4.58	**2.5**	1.40-4.50
All Protozoa	**7.8**	5.73-10.61	**11.8**	9.13-15.11	**6.4**	3.89-10.33	**7.2**	4.51-11.37	**9.7**	7.23-12.93
*B. hominis*	**3.4**	2.11-5.41	**5**	3.33-7.38	**2.5**	1.11-5.48	**2**	0.79-4.9	**3**	1.69-5.01
Non-pathogenic										
Amoebae	**2.9**	1.72-4.82	**6.4**	4.42-8.99	**2.3**	0.96-5.19	**2.5**	1.08-5.47	**3**	1.69-5.01
*G. duodenalis*	**2**	1.04-3.63	**3.2**	1.88-5.19	**1.4**	0.43-3.91	**2**	0.79-4.90	**3.4**	2.02-5.52

(Date based on new arrivals and residents combined)

### Species richness and combinations of species

Fifty one subjects carried more than one species of parasite (3.3% [2.47-4.36] of the whole study group), most of these (*n *= 49) were infected with 2 species combinations, only two individuals had 3 species recorded and no-one was recorded as infected with more than three species. One of the 3-species combinations involved hookworms, *A. lumbricoides *and *T. trichiura*, and the other just protozoan species (non-pathogenic amoebae, *G. duodenalis *and *B. hominis*).

With 12 species of parasites (helminth and protozoan species) 66 different combinations of pair-wise combinations are possible, but only 21 of these were observed. Among subjects with helminths alone, all pair-wise combinations involved one of the common soil-transmitted helminths (hookworms, *A. lumbricoides *or *T. trichiura*), and in 13 of the 18 subjects with these combinations, one of the species was a hookworm. Similarly, among the 19 subjects who had 2 species combinations involving both a helminth and a protozoan species, 14 had hookworms. Where 2-species combinations involved only protozoan parasites (*n *= 12), one of these was always the non-pathogenic amoebae.

When analysis was confined to the subjects carrying multiple infections, to determine if multiple infections were focused in any particular group in the analysis (region, residency, sex and age class), there was a significant sex-dependent effect of region of origin (region of origin * sex, χ^2^_4 _= 19.2, *P *= 0.001). Multiple infections were more common among male Nepalese and male W. Asians (21.6% of all multiple infections [14.41-30.87] in each case), rare (2.0% [0.34-7.26]) among female W. Asians, and non-existent among female N. Africans and female Nepalese (0% [0-3.82] in each case). Multiple infections were perhaps not surprisingly more common among males, whether resident (49.0% [38.90-59.13]) compared with resident females (3.9% [1.32-9.81]) or recent immigrants males (25.5% [17.68-35.02]) versus recent immigrant females (21.6% [14.41-30.87]). This difference between groups was significant when confined to the subjects with multiple infections (for residency status*sex χ^2^_1 _= 10.5, *P *= 0.001). Male Nepalese residents accounted for 17.6% (11.07-26.63) of the 51 multiple species infections, male W. Asians residents for 15.7% (9.52-24.19) and female immigrant N.E. Africans for 11.8% (6.61-19.73).

When the frequency distribution of the number of parasitic species harboured by people was compared to the null model of Janovy [7], the observed data were found to differ significantly from the null model (χ^2^_3 _= 12.6, *P *= 0.0056). Thus, in the absence of focused occurrence or interactions between species, the null model predicted 35, compared with our observed 49 cases of 2-species infections, and more single species infections (316 compared with our observed 279). The prediction of the number of 3-species infections was entirely in line with our observations (*n *= 2 in both case).

### All helminths combined

**Figure 1:**
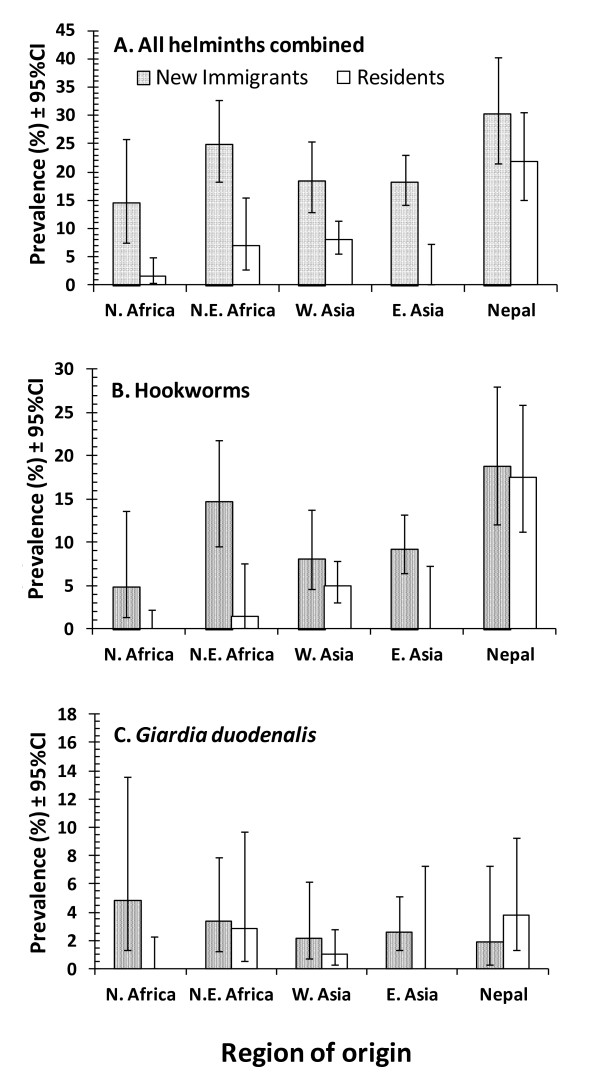
**Variation in the prevalence of helminths**. Variation in the prevalence of helminths (all species combined) (A), hookworms (B) and *Giardia duodenalis *(C) by region of origin of subjects and residency status. The minimum sufficient models for A, B and C were LR χ^2^_158 _= 133.3 (*P *= 0.9), LR χ^2^_158 _= 111.1 (*P *= 0.998), and LR χ^2^_152 _= 95.7 (*P *= 1.0), respectively. All models included additional terms which did not incorporate prevalence of parasites. For significance of the illustrated effects see text.

The overall prevalence of helminths in the study group was 14.6% (Table 2). Prevalence was highly affected by residency status but was dependent also on region of origin (region of origin*residency status*presence/absence of helminths, χ^2^_4 _= 16.8, *P *= 0.002). The data in Figure 1A show that in subjects from each of the five regions, the prevalence of helminths was higher among recent immigrants compared with residents. The discrepancy between residents and recent immigrants was perhaps greatest among those from N. Africa and E. Asia, and least among those from Nepal, among whom residents showed far higher levels of prevalence than those from any of the other four regions. As with all parasites combined, prevalence of helminths was also highly dependent on host sex (sex*residency status*presence/absence of helminths, χ^2^_1 _= 9.9, *P *= 0.002). Among recent immigrants prevalence of helminths was high and very similar in both sexes (females = 21.2% [16.63-26.47] and males = 18.8 [14.44-23.94]). However significant sex-based differences were detected among residents; females showed a very low prevalence (1.3% [0.26-5.06]) whilst prevalence was 8.3 fold higher in males (11.1% [9.10-13.42]). To a large extent this sex difference was driven by the high prevalence of helminths among males from Nepal (of 71 helminth positive residents, 69 were male and among these 58% were Nepalese, the rest coming mostly from bordering countries in the Indian subcontinent [9% from Bangladesh, 23% from India, 3% from Sri Lanka]) and only 7% from other regions (4% from Ethiopia and 3% from Egypt).

### Individual species of helminths

The most frequently encountered helminths were hookworms (Table 2). Analysis showed that they varied significantly between regions of subject origin but this also depended on whether they were new immigrants or residents (region of origin*residency status*presence/absence of hookworms, χ^2^_4 _= 15.13, *P *= 0.004). Table 2 shows that the overall prevalence was higher among new immigrants. While this was true for N. and N.E. Africans, and for W. and E. Asians to varying degrees, it was not true for the Nepalese, among whom the prevalence of hookworms was very similar irrespective of whether they were new immigrants or residents (see Figure 1B). The prevalence of hookworms also varied between the sexes based on residency status (region of origin*sex*presence/absence of hookworms, χ^2^_1 _= 4.1, *P *= 0.004). Prevalence was very similar in male (9.4% [6.39-13.52]) and female (11.3% [7.91-15.62]) new immigrants and stayed high among male residents (7.6% [5.91-9.58]) but dropped markedly among resident females (0.7% [0.08-3.93]).

*T. trichiura *was the second most common nematode in the study group (Table 2). Prevalence was 6-fold higher among new immigrants, but this was also dependent on host sex (residency status*sex*presence/absence of *T. trichiura*, χ^2^_1 _= 4.5, *P *= 0.034). In female subjects there was a 7-fold difference, with prevalence dropping from 7.1% [4.54-10.78] in new immigrants to 0% [0-2.81] among residents; in male subjects prevalence dropped from 4.9% [2.91-8.22] to 1.3% [0.71-2.32]. Despite the higher prevalence for E. Asians (Table 3), there was no overall difference between regions of origin.

The third most common intestinal nematode, *A. lumbricoides *was 5 fold more frequently encountered among the new immigrants compared with residents (Table 2, residency status*presence/absence of *A. lumbricoides*, χ^2^_1 _= 12.2, *P *< 0.001). This contrast in prevalence between new immigrants and residents was observed in both sexes (in females from 2.9% to zero, and in males from 3.6% to 0.8%), so there was no significant difference between the sexes in this respect, nor in overall prevalence (males = 1.9% and female 2.1%). Prevalence also varied significantly between the age classes (age*presence/absence of *A. lumbricoides*, χ^2^_6 _= 16.9, *P *= 0.01), but there was no distinct trend in relation to increasing age apart from the highest prevalence being recorded in age class 1 (6.0% [2.76-12.0]) before falling to lower values in all the older classes (range 0.8% - 2.9%). There was no significant difference between regions of origin, prevalence varying from 1.6-2.5% across the 5 regions (Table 3).

The factors affecting prevalence of the remaining helminths listed in Table 2 were not analysed because overall prevalence was at most 1.0% and mostly lower for these species.

### All protozoa combined

The overall prevalence of protozoan infections was lower than that for helminths (Table 2). Only one significant effect emerged from the analysis, which was a difference in the pattern of prevalence across the age classes in the five regions (region of origin*age class*presence/absence of helminths, χ^2^_24 _= 44.6, *P *= 0.006). Close scrutiny, however, did not reveal any clear underlying trend. As the data in Table 2 show, overall prevalence of intestinal protozoan infections was very similar among residents and among recent immigrants. However, these figures, derived from combining presence/absence of all the individual species, hid underlying differences between the two categories among individual species (see below).

### Individual species of protozoa

The most common protozoan taxon was that for non-pathogenic amoebae. The prevalence values for residents and recent immigrants (Table 2) were not significantly different. There was no sex effect (prevalence in males = 3.3% [2.26-4.61] and in females = 2.8% [1.93-4.10]), and this was true in both newly arrived immigrants and residents. Prevalence did not vary significantly between the regions, although as Table 3 shows, prevalence was numerically higher among N.E. Africans; however, this was confounded by a significant interaction with age (age*region of origin*presence/absence of non-pathogenic amoebae, χ^2^_24 _= 42.1, *P *= 0.013), although no clear age-related pattern was evident.

The second most common species of protozoan was *B. hominis*. In contrast to all other species, this parasite was more common among residents (Table 2; residency status*presence/absence of *B. hominis*, χ^2^_1 _= 27.7, *P *< 0.001) and a similar pattern was observed among both male (1.0% and 5.5%) and female (0.5% and 3.3%) new arrivals and residents, respectively. There was also a significant independent effect of host sex (sex*presence/absence of *B. hominis*, χ^2^_1 _= 6.2, *P *= 0.013), *B. hominis *being almost three times as common in males (prevalence = 3.8% [2.67-5.18]) compared with females (prevalence = 1.3% [0.77-2.26]). Prevalence also varied significantly between regions (region of origin*presence/absence of *B. hominis*, χ^2^_4 _= 14.3, *P *= 0.006), being lowest among E. Asians and highest among N.E. Africans (Table 3). Closer examination of the data revealed that prevalence among residents was particularly high among both male and female N.E. Africans (18.8% and 7.7%, respectively). In fact, resident males from Sudan and Ethiopia both showed high prevalence (18.2% and 20.0%), as did female residents from Ethiopia (14.3%) but not resident female Sudanese (4.0%). For *B. hominis*, then, a N.E. African origin for residents was clearly a risk factor, but interestingly this was not the case for recent arrivals from this region among whom prevalence in females (2.0%, *n *= 101) and males (0%, *n *= 48) was considerably lower. Overall, in male subjects from all 5 regions the prevalence of *B. hominis *was higher among residents compared with new arrivals, and among female subjects this was the case in 3 regions (N. Africans, N.E. Africans and W. Asians). However, this was not the case in E. Asians or the Nepalese; neither female new arrivals nor female residents from these regions were infected with this parasite.

In terms of frequency of occurrence, *G. duodenalis *was the third most common species (Table 2). Numerically, prevalence was higher among new immigrants compared with residents as the data in Table 2 show, but this difference between residency status groups was also confounded by a sex effect (residency status*sex*presence/absence of *G. duodenalis*, χ^2^_1 _= 3.85, *P *= 0.05). Thus among new immigrants prevalence was 3.1% (1.58-5.97) in males and 2.6% (1.25-5.33) among females. In male residents prevalence was lower compared to both sexes of new immigrants (1.9% [1.18-3.13]) but this parasite was absent from the 150 resident women that were assessed (0% [0-2.81%]). Prevalence also varied significantly depending on the region of origin (Table 3) but this was dependent on residency status (Figure 1C; residency status*region of origin*presence/absence of *G. duodenalis*, χ^2^_4 _= 11.6, *P *= 0.02). Figure 1C shows that new immigrants from four of the regions had higher prevalence than residents (in fact residents from N. African and E. Asian had no *G. duodenalis *infections at all), but among the Nepalese, prevalence was numerically higher among residents. There were also significant differences between the age classes (age *presence/absence of *G. duodenalis*, χ^2^_6 _= 12.7, *P *= 0.047), with the younger subjects (age classes 1, 3 and 4) generally showing higher prevalence than the older ones (5, 6 and 7).

The factors affecting prevalence of the disease causing *E. histolytica *were not analysed because overall prevalence at 0.8% (Table 2) was too low for meaningful interpretation.

## Discussion

Utilising hospital-based analyses of stool samples from recent arrivals and resident foreign immigrant workers in Qatar, this study has confirmed findings from earlier studies that the prevalence of many intestinal parasitic infections is higher among those recently arrived in Qatar, compared with long-term residents. This finding provides support for the policy of treatment for helminth infections with albendazole on arrival. Nevertheless, it is worth emphasizing that single dose 400 mg albendazole is not very effective against *T. trichiura *[8] and although it has some effect against *G. duodenalis *[9] it probably does not affect the other protozoan infections. No antiprotozoal treatment is given routinely to new immigrants. The reduction in prevalence of *T. trichiura *and pathogenic amoebae among residents compared to new immigrants must therefore be attributable to other factors, possibly improvement in sanitation relative to conditions at home and improved health care. Whilst it is reassuring that prevalence rates are generally low, it is of concern that some infections persist among long-term residents in Qatar, despite the fact that sanitation is generally of an adequate standard, tap water is considered to be potable and medication is easily obtainable. All piped water in Qatar comes from desalination plants, and hence, unlike river water in the tropics, this is unlikely to be a source of infection. However, as pointed out earlier [5] the persistence of water borne intestinal protozoan infections suggests that water supplies become contaminated at some point downstream between desalination and consumption, or that locally, the faecal-oral route of transmission of these pathogens is facilitated in some other way.

In our earlier analysis of intestinal parasitic infections among residents [5] we had found evidence of rising prevalence of helminth infections in the period 2005-2008. For example the prevalence of all helminths combined rose from 1.3% among residents in 2005 to 4% in 2008. In the present work based on data collected a year later in 2009, the prevalence was even higher at 14.6%, and although this is of concern, it is important to realise that the two datasets are not directly comparable, because for the present analysis in order to obtain balanced datasets we concentrated on subjects from countries of origin that were represented both among the newly arrived immigrants and among the resident population. Thus from the originally collected comprehensive dataset, 468 records were removed, including those of 258 Qatari nationals (by definition can only be residents) whose inclusion would have reduced overall prevalence rates. Another limitation of the current work is our reliance entirely on prevalence calculated from presence/absence data. While perhaps limiting epidemiologically, these are the only routinely collected data held by the institutes that provided us with records. The routine assessment of intensity of infection with intestinal parasites by methods such as the Kato-Katz or McMaster, would have provided greater depth to the analyses but when very large numbers of patients are involved, this is expensive and in competition with other priorities not cost-effective, as in this case. Nevertheless, this deficiency in our approach in the current study, and in our earlier papers [4,5] will be addressed in future work in the region, through which we intend to probe further the underlying causes of the trends we have reported here.

Of particular significance was our finding that among the Nepalese immigrants the prevalence of helminth infections was high in both newly arrived individuals [10] and among settled long-term residents, the latter having already been identified as a risk factor in earlier published studies [5]. The similarity in prevalence between these two groups among the Nepalese contrasted markedly with all other regional groups (see Figure 1), where prevalence was markedly higher among newly arrived immigrants compared to residents. The possible explanation for such high prevalence and persistence of helminths among the resident Nepalese workers most likely lies in frequent return visits by workers to their village homes in Nepal [5] where helminth infections are widespread [11] and/or local transmission in Qatar in the poorer quarters of the city where the Nepalese tend to congregate. However, because of Qatar's extremely hot and arid climate, it was felt unlikely that the transmission stages of GI nematodes, such as hookworms in particular (the principal species harboured by the Nepalese), can survive long enough in the environment to enable infective stages to develop in Qatar, so this persistence of intestinal helminth infections among the Nepalese, mostly young adult males, still remains to be fully explained.

Another related trend in the data-set that is worth commenting on is the contrast in prevalence of infections between the sexes among the resident population, relative to new immigrants. Thus among new immigrants the prevalence rates were very similar between the sexes when all parasites were combined, all helminths or all protozoa were considered. Sex differences only really became apparent among the residents and when region of origin was collapsed, among residents there was clear male bias with, for example, prevalence of helminths being over 8.3-fold higher among males. This was particularly evident also for hookworm and *T. trichiura*, but also for the protozoan *G. duodenalis *infections. Consistent with earlier reports [5] and based on analysis of earlier data from the period 2005-2008, in the case of helminths this sex difference among residents was largely driven by the high prevalence among resident males from Nepal, especially with respect to hookworms (Prevalence among male Nepalese immigrants = 19.6% and among residents = 17.7%). On a broader front, another contributory factor may lie in many of the immigrant female workers taking up jobs in the catering/food handling industries or as house maids [4], where perhaps greater attention is given to antiparasite therapy (for food handlers annual inspections are mandatory) and prophylaxis, whereas male immigrants live in labour camps in poorer quarters of the cities, mostly join the labour force and perhaps are less attentive to their health needs. Interestingly, in marked contrast to the Nepalese, among whom prevalence hardly declined after acquiring residency status, N.E. Africans arrive in Qatar with prevalence of hookworms only secondary to that of the Nepalese (13.9% for females and 16.7% for males and see Figure 1B for combined values) but in both cases lose the worms as residents.

*B. hominis *was three times more common overall among male subjects but in this species there were few cases among new arrivals. Prevalence then rose in both sexes, and to a similar extent, among the residents compared with new arrivals (six-fold increase overall), a pattern contrasting with all other infections, and the highest prevalence was recorded among subjects with a N.E. African origin. This was particularly true among Ethiopians, among whom both resident sexes showed higher prevalence compared with new arrivals. The overall prevalence of *B. hominis *was 5.1% among residents, similar to the 4.3% reported previously [5]. However, the relatively low prevalence of just 0.8% among new arrivals, contrasted with the earlier reported values, which ranged from 9.5% to 16.4% among female new immigrants in 2005 and 2006 [4]. It is of interest that in this same report, male newly arrived immigrants who were seeking work in food handling, had a prevalence of *B. hominis *of only 1.3% in 2006. Nevertheless, the current analysis indicates that newly arrived subjects from all regions generally show a low prevalence of this protozoan. However, they become infected during residency, and Ethiopians and Sudanese seem particularly prone to acquiring infection with this parasite while, or after, acquiring residency status, but whether this is because of genetic or environmental factors remains to be explained.

The exceptions to sex-biased prevalence were *A. lumbricoides*, and non-pathogenic amoebae. *A. lumbricoides *showed similar very low prevalence in both sexes of residents, lower in both cases compared to new arrivals. In the case of non-pathogenic amoebae, there was also a minor drop in females but a comparable increase among males, but in neither sex was the change marked.

The prevalence of *G. duodenalis *in this study was comparable to levels found among residents in 2005-2008 (1.6% here and 1.9% in the earlier study [5]). Prevalence among newly arrived immigrants was higher at 2.9% although not as high as levels reported earlier [4], for newly arrived food handlers and house maids (ranging from 1.3% among males to 8.6% among females in 2006). The continuing presence of both *B. hominis *and *G. duodenalis *among the resident population in Qatar, the latter especially among the Nepalese, suggests that transmission is taking place locally; since both are generally regarded as water-borne pathogens, the water distribution system and waste disposal systems in Qatar are likely to be implicated. Poor sanitation and hygiene may also enable transmission by the faecal-oral route in both cases. *G. duodenalis *is an important disease causing pathogen, considered to be among the leading agents responsible for childhood diarrhoea [12] but *B. hominis *is generally regarded as innocuous [13,14]. However, this latter species can cause chronic and recurrent infections in weaker patients such as the elderly or immunocompromised [15,16] and recent studies have indicated that it is better considered as a group of genetically diverse organisms, among which some genotypes can also cause disease in immunocompetent humans [17].

## Conclusions

The contrasting patterns of infection that we detected among recent immigrants and residents in Qatar are intriguing and current research is focusing on the underlying causes. Clearly the Nepalese immigrants and particularly those who take up residency in Qatar require special attention from the health authorities and epidemiologists/social scientists to unravel why intestinal helminths, particularly hookworms, and *G. duodenalis *persist among them after the acquisition of residency status. The general increase in *B. hominis *infections in all groups among residents, but particularly the unusual susceptibility of N.E. Africans from Ethiopia and Sudan to *B. hominis *infection, is also of interest, and one avenue that must be explored in the near future is whether transmission is water borne, or whether other routes of infection perpetuate this species in Qatar, especially among N.E. Africans.

## List of abbreviations used

*n*: number of subjects in sample; LR: likelihood ratio; GLM: general linear model.

## Competing interests

The authors declare that they have no competing interests.

## Authors' contributions

MAAM conceived the study, collected the data, and wrote the manuscript. JMB analysed the data and wrote the manuscript. AI participated in the conception of the project, helped in data collection, interpretation of data and drafting of the manuscript. NA and KH carried out the stool examination. RE carried out the stool examination and confirmed the identity of the parasites. All authors read and approved the final manuscript.
